# Supplementary material for: A diminutive new basilosaurid whale reveals the trajectory of the cetacean life histories during the Eocene
Source: Commun Biol. 2023 Aug 10;6:707. doi: 10.1038/s42003-023-04986-w (PMC10415296; doi:10.1038/s42003-023-04986-w)
Supplement: Supplementary file 6 — Supplementary Data 3 [file 42003_2023_4986_MOESM6_ESM.zip › Supplementary Data 3/Supplementary Data 1_BTD_ASR/trait_0155_tree.plot.pdf]

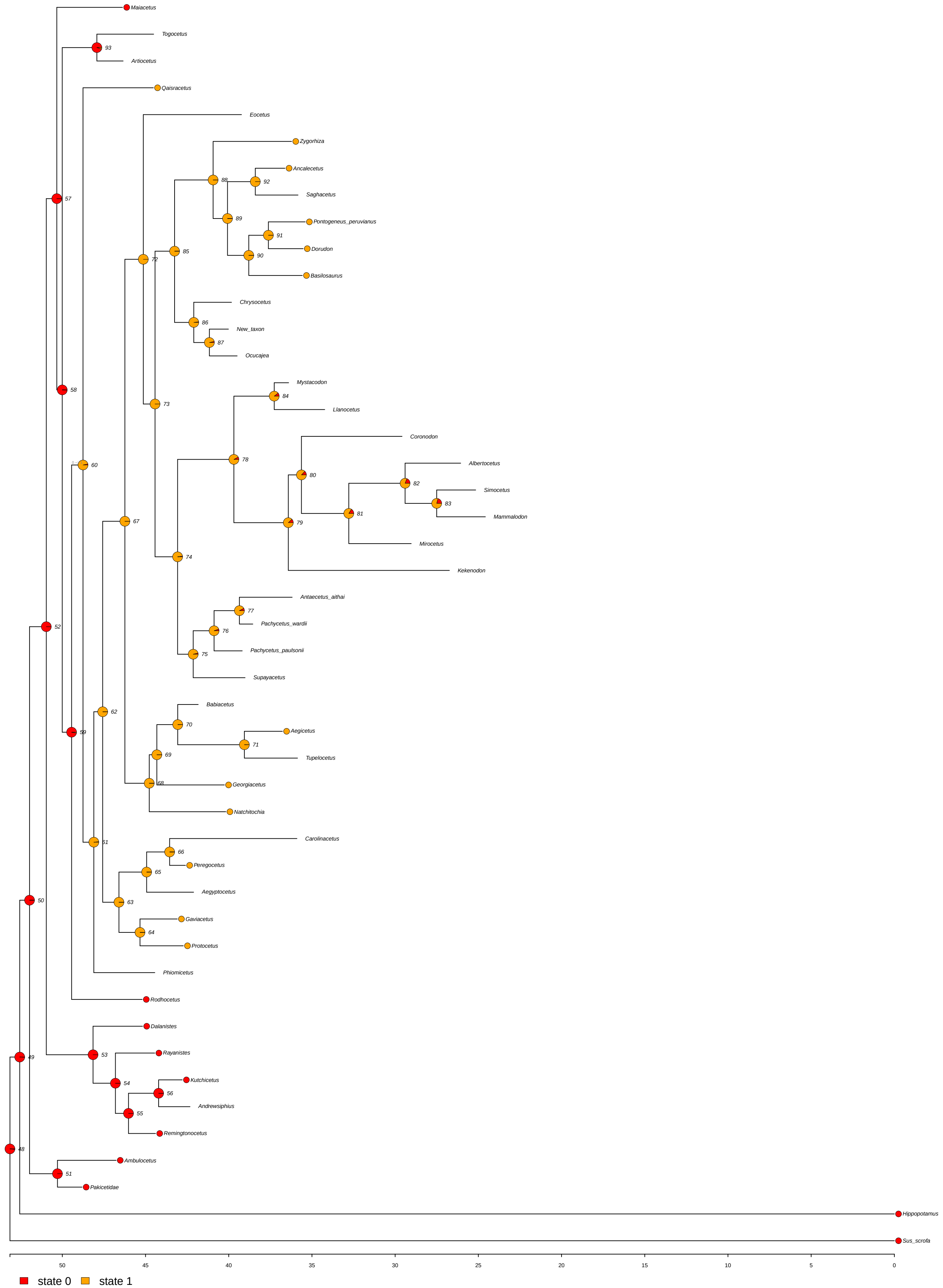

Maiacetus
Togocetus
93
Artiocetus
Qaisracetus
Eocetus
Zygorhiza
Ancalecetus
88
92
Saghacetus
57
Pontogeneus_peruvianus
89
91
90
Dorudon
72
85
Basilosaurus
Chrysocetus
86
New_taxon
87
Ocucajea
Mystacodon
84
58
73
Llanocetus
Coronodon
60
78
80
Albertocetus
82
Simocetus
83
67
81
Mammalodon
79
Mirocetus
74
Kekenodon
Antaecetus_aithai
77
Pachycetus_wardii
76
52
Pachycetus_paulsonii
75
Supayacetus
Babiacetus
62
70
Aegicetus
59
71
Tupelocetus
69
68
Georgiacetus
Natchitochia
61
Carolinacetus
66
Peregocetus
65
Aegyptocetus
50
63
Gaviacetus
64
Protocetus
Phiomicetus
Rodhocetus
Dalanistes
53
Rayanistes
49
54
Kutchicetus
56
Andrewsiphius
55
Remingtonocetus
48
Ambulocetus
51
Pakicetidae
Hippopotamus
Sus_scrofa
50
45
40
35
30
25
20
15
10
5
0
state 0
state 1
